# Soft tissue considerations in modern restorative dental practice

**DOI:** 10.6026/973206300223652

**Published:** 2026-06-30

**Authors:** Fatima Sarwar, Osiris Toledo Calixto, Nisha Dholakiya, Ramin Torkizadeh, Anuja Chandra, Gandharvi Kakarla

**Affiliations:** 1 Department of Dentistry, University of Lahore, Lahore, Pakistan; 2 Department of Stomatology, Benemerita Universidad Autonoma de Puebla, Puebla, Mexico; 3 Department of Dentistry, College of Dental Science and Research Center, Ahmedabad, Gujarat, India; 4 Department of Dentistry, University of Liverpool, Liverpool, UK; 5 Department of Dentistry, KLE Institute of Dental Sciences, Belgaum, India; 6 Department of Dentistry, Dr. NTR University of Health Sciences, Vijayawada, India

**Keywords:** Tissue management, supracrestal tissue, gingival retraction, retraction advances, tissue integrity

## Abstract

In dentistry, the success of a restoration depends not only on the choice of material or technique but also on the preservation of periodontal physiology and health. A healthy periodontium forms the essential foundation for long-term restorative success, making an interdisciplinary approach indispensable. Respecting biologic width is critical to prevent inflammation and attachment loss, while non- surgical methods such as provisional contouring can effectively guide soft tissue healing. Surgical strategies, including crown lengthening, are employed to correct deficiencies and establish harmony between esthetics and function. In complex restorative situations, gingival margin elevation requires precise planning to balance tissue stability and restorative demands. Patient-related factors, such as gingival biotype and healing capacity, further influence tissue behavior and outcomes. Ultimately, careful management of both soft and hard tissues ensures predictable and durable prosthodontic results.

## Background:

Restorative dentistry focuses on bringing back the normal health, appearance and function of natural teeth while keeping the gums healthy and balanced. The long-term success of any dental restoration depends on how well the surrounding gum tissues stay stable and healthy [1]. Good soft tissue management helps prevent problems like inflammation, gum recession and tissue loss. Today, periodontal and mucogingival procedures are designed to meet both functional and esthetic needs, leading to better restorative results [2]. These improvements come from a better understanding of how tissues heal and the use of modern biomaterials that support long-term stability [3]. Therefore, it is of interest to describe the management of soft tissue effectively in restorative dentistry.

## Biological concepts in soft tissue management:

Restorations must respect soft tissue biological parameters to preserve periodontal health and prevent gingival inflammation or bone loss [4]. One key factor influencing gingival response is the position of restorative margins, which should ideally remain supragingival to facilitate plaque control and maintain gingival health. When esthetic or structural demands require, subgingival margins, smooth surface finishing and respect for the epithelial and connective tissue attachments become essential [5]. The concept of biologic width, defined as the combined epithelial and connective tissue attachment above the alveolar bone, plays a critical role in maintaining gingival integrity [6]. Disruption of this zone, such as by over extended crown margins, can trigger chronic inflammation, crestal bone loss and attachment breakdown. Corrective procedures such as crown lengthening or orthodontic extrusion may be necessary to restore a proper biologic width and reestablish gingival health [7]. During tooth preparation, the dentist must avoid trauma to the junctional epithelium, connective tissue fibers, or crestal bone, as mechanical or chemical injury may lead to tissue recession and compromised restoration margins. An in-depth understanding of how restorative designs influence peri-restorative tissues enables clinicians to achieve both esthetic predictability and biological compatibility over time [8].

## Biologic width and its role in restorative dentistry:

The term supracrestal tissue attachment (STA) has replaced the older term biologic width (BW). Meaning the two soft tissue layers that connect the tooth to the gum the junctional epithelium and the connective tissue located above the alveolar bone crest. This updated terminology was introduced in 2017 during the World Workshop on the Classification of Periodontal and PeriImplant Diseases and Conditions, organized by the American Academy of Periodontology (AAP) and the European Federation of Periodontology (EFP), replacing the 1999 classification. In restorative dentistry, preserving the STA/BW is essential to promote and maintain proper function and periodontal health. Although individual variation exists, it is generally accepted that at least 3 mm should separate the alveolar crest from the restoration margin. This space includes approximately 2 mm for the STA/BW, which means the attached gingiva must be wide enough around the abutment [9]. Identifying the crest category through bone sounding is key. This evaluation has a significant impact on gingival health, esthetics and function. Bone sounding measures the distance from the gingival margin to the alveolar crest. It helps classify the crest as normal, high, or low, which may be stable or unstable [10]. This assessment also determines whether the restoration margin invades the biological width. Such an invasion can compromise soft tissue health and long-term restorative outcomes. Deep placement promotes bacterial accumulation and tissue damage, which can compromise the long-term success of the restoration [11]. Common options for margin placement of restorations are: supragingival, equigingival and subgingival. Among them, subgingival margins are the most likely to cause complications such as open margins, overhangs and over-contoured restorations. The greatest biological risk occurs when margins are placed too deep into the sulcus or when the STA/BW is violated [12 , 13].

## Clinical Signs of STA/BW Violation [11]:

[1] Chronic inflammation around the restoration

[2] Bleeding on probing

[3] Localized hyperplasia

[4] Gingival recession

[5] Pocket formation

[6] Clinical attachment loss

[7] Alveolar bone loss

## Evaluation of biologic width in restorative dentistry:

[1] Clinical methods: Probing sulcus depth, tissue rebound after preparation and visual inflammation [12].

[2] Radiographic evaluation: Distance from restorative margin to alveolar crest (should be ≥2 mm). Traditional X-rays lack 3D detail and can produce distorted or overlapping images, making it difficult to precisely measure the biologic width, especially in the interproximal areas of posterior teeth.

[3] CBCT evaluation: 3D assessment of bone and tissue relationships when available. CBCT offers superior diagnostic detail and accuracy for evaluating biologic width, especially in complex cases that require precise surgical/restorative planning.

Recommendations to protect the biologic width in restorative dentistry [9, 14]:

[1] Avoid placing the margin too deep into the sulcus during tooth preparation.

[2] Use proper gingival retraction to ensure an accurate impression.

[3] Prevent over-contouring, as it can lead to plaque accumulation and gingival inflammation.

[4] Select the pontic design according to esthetic demands and cleaning accessibility, since it directly impacts gingival health.

[5] Pay special attention to soft and hard tissue in all phases of restorative treatment to predict a successful outcome.

## Non-surgical approaches to soft tissue management in restorative dentistry:

Effective soft tissue management is essential in restorative dentistry to ensure optimal restorative outcomes. Non-surgical techniques emphasize gingival isolation and retraction for hemorrhage control and temporary tissue displacement while maintaining respect for the biologic width. Mechanical approaches include the use of rubber dams, gingival clamps and retraction cords that may be braided, twisted, or knitted. Cord packing remains the most common, rapid, simple and cost-effective method for gingival retraction [15].

## Cord packing in gingival retraction:

The placement of cord packing may be performed with or without hemostatic agents using single- or dual-cord techniques. In the dual-cord technique, a thinner apical cord is left in place during impression making to delay tissue rebound, while a thicker coronal cord provides mechanical displacement. This approach affords better access and bleeding control but may cause discomfort and unpredictable tissue resorption, making it unsuitable for supra-gingival margins. The single-cord technique is simpler but risks tissue collapse in deep sulci, compromising impression accuracy [16].

## Limitations and considerations:

Cord packing can cause transient inflammation or sulcular trauma; however, healing usually occurs within two weeks. The method demands considerable clinical skill, as improper placement can result in gingival damage or retained cord fibers. Nonmedicated cords are biocompatible but offer limited hemostatic control; moistening them before placement can minimize bleeding risk. Optimal impressions require a sulcus width of at least 0.2 mm and a placement time of four minutes or longer. Serrated instruments are preferred for braided cords, while the non-serrated types are suitable for twisted cords. Copper-reinforced cords can facilitate easier placement [17].

## Chemical methods of gingival retraction:

Chemical retraction agents support sulcular expansion and bleeding control and are often used along with mechanical approaches [15]. These agents include vasoconstrictors, hemostatic compounds and astringents. Vasoconstrictors such as epinephrine, tetrahydrozoline and oxymetazoline constrict blood vessels, reducing bleeding and transiently retracting tissues. Astringents act by precipitating tissue proteins, decreasing capillary permeability and strengthening the mucosal surface. Aluminum chloride is widely preferred for its minimal irritation and effective sulcus maintenance [20]. Although epinephrine provides potent hemostasis, it carries systemic risks such as tachycardia and hypertension and is contraindicated in patients with cardiovascular conditions. Safer alternatives such as tetrahydrozoline produce adequate retraction with fewer adverse effects [16]. Mechanico- chemical techniques use cords impregnated with aluminum chloride or ferric sulfate to enhance tissue displacement and hemostasis, but care is required to prevent tissue damage and postoperative sensitivity. Hemostatic agents control active bleeding from arterioles and capillaries, facilitating moisture control during restorative procedures. However, residues can interfere with impression accuracy if not rinsed thoroughly. Ferric sulfate offers rapid hemostasis but may cause tissue discoloration and compromise bonding [17]. Ferrous sulphate can similarly stain tissues and restorations. Safer alternatives include alum and aluminum sulfate, which provide mild retraction with minimal irritation, while tannic acid ensures gentle hemostasis and favorable tissue recovery. Zinc chloride is discouraged due to its caustic potential and Negatol solution is avoided because of its strong acidity and possible enamel damage. Cordless systems, such as GingiTrac, Expasyl and Magic Foam Cord, utilize expanding pastes or foams that atraumatically widen the sulcus while controlling bleeding, typically with the adjunct use of compression caps [18].

## Surgical approaches to soft tissue management:

While non-surgical gingival displacement methods remain common in restorative dentistry, certain clinical scenarios demand surgical approaches for optimal margin exposure and moisture control. These techniques, through more invasive, can offer enhanced precision and predictable soft-tissue contours when performed correctly [19]. Troughing or tissue dilation is a procedure used to reduce gingival enlargement and expose the gingival margins. The gingival trough created by soft-tissue excision typically runs from the crest of the free gingival margin to about 0.3-0.4 mm apical to the finish line of the tooth preparation. Unlike non-invasive displacement methods, surgical approaches physically remove a band of gingival tissue, which then must undergo a brief healing phase before final restorative procedures [20]. Rotary gingival curettage exemplifies another surgical approach. Using high-speed rotary burs, the clinician carefully sculpts a trough in the sulcus around the preparation's finish line, removing the inner epithelium and part of the connective tissue. Local anesthesia is essential to ensure patient comfort and minor bleeding at the site is expected [21]. Laser means-Light Amplification by Stimulated Emission of Radiation ||. Lasers are classified by their method, which includes the type of medium used (gas laser or solid laser), the application (hard or soft tissue) and the wavelength. In dentistry, its application is categorized into soft tissue or hard tissue applications [22]. In restorative dentistry, soft-tissue lasers excel at moisture control and hemostasis, making them especially valuable for precise gingival management. Whether trimming tissue for aesthetic crown lengthening, excising overgrowth, or creating a trough around a preparation, they deliver clean, controlled cuts with fewer biological side effects. Although not yet universal in every practice, these lasers show clear promise for improving patient comfort and clinical outcomes. Soft-tissue lasers provide the operator with enhanced control and minimal impact on the surrounding tissue. Diode lasers, in particular, emit wavelengths preferentially absorbed by gingiva without affecting enamel, dentin, metal alloys, or porcelain [21]. Electrosurgery is a method in which a high frequency electrical current passes through tissue. This small electrode is aligned with the tooth's long axis to widen the gingival sulcus, removing tissue solely from its inner wall. Electrosurgical techniques are valued for their ability to produce rapid, clean incisions with effective hemostasis, though operator control is essential to prevent excessive thermal injury to adjacent tissues [23].

## Recent advances in gingival margin management:

Now that all the methods of achieving optimal restorative outcomes via gingival marginal elevation are described above, this section highlights the recent trends. These established techniques, when selected and applied appropriately, offer predictable control, minimal trauma and enhanced healing. Emerging innovations now push boundaries, refining precision and elevating soft tissue management [26].

## Recent advances:

Recent clinical studies emphasize that gingival phenotype plays a decisive role in soft-tissue stability around restorative margins,with thicker biotypes demonstrating more predictable healing and reduced inflammatory response following restorative procedures [24].GingiTrac™is a vinyl polysiloxane (VPS)-based, cordless gingival retraction material designed to simplify and accelerate sulcular displacement for both single and multiple crown preparations. Since its introduction, it has gained recognition for combining built-in astringency with predictable, atraumatic tissue management. It features a medium-viscosity VPS formula infused with 15% ammonium aluminium sulphate. Upon expression into the gingival sulcus, the material exerts gentle, uniform pressure to displace marginal tissues while the alum component provides hemostasis by controlling crevicular fluid and minor bleeding. As a silicone- based paste, it sets in approximately two minutes, after which it can be removed intact, leaving a dry, well-retracted sulcus ready for impression taking.

## It comes in two types of delivery systems:

[1] MiniMix System: A compact, patient-friendly syringe and mixing tip ideal for accessing posterior zones with minimal effort [25].

[2] Automix Cartridges: Standard 50 mL cartridges paired with Super Mixer™ nozzles to reduce waste by 33%, yielding up to ten additional applications per cartridge [26].

Racegel is a thermo sensitive gingival retraction gel formulated with 25% aluminium chloride. At room temperature, it remains a low- viscosity liquid for easy injection; upon contact with warm tissues, it rapidly transforms into a gel that exerts gentle, uniform pressure to open the sulcus and control bleeding [28].Delivered in ready-to-use syringes with fine, pre-bent needle tips, the bright orange color ensures precise placement without dripping. After two minutes of action, a cold-water rinse re- liquefies the gel for effortless removal, leaving no rebleeding or staining. By creating just enough displacement for accurate analog or digital impressions and eliminating the need for cords or separate hemostatic agents-Racegel offers a fast, atraumatic solution that enhances patient comfort and streamlines restorative procedures [29].Expasyl Retraction System is injectable, aluminium chloride-based retraction paste introduced in 2000. Formulated with 15% aluminium chloride, it combines ideal viscosity and hemostatic action to displace the sulcular epithelium atraumatically. In practice, the paste is syringed into the gingival sulcus for 20 seconds, allowed to act for 1-2 minutes and then rinsed off, providing simultaneous tissue retraction and hemostasis and crevicular fluid control. It generates approximately 143 kPa of retraction pressure-37 times lowerthan that produced by cords minimizing the risk of epithelial detachment or tissue trauma [27].By stabilizing and dehydrating the sulcus in less than two minutes, it also facilitates precise analog and digital impressions critical for CAD/CAM workflows [26].

## Patient factors influencing soft tissue outcomes in restorative dentistry:

The long-term success of restorative treatment is shaped not only by restorative materials and operative techniques but also by patient- specific factors that influence peri-restorative soft tissue health. Systemic conditions, medications, salivary function, oral hygiene and smoking can significantly affect tissue response, healing and long-term stability [30].

## Systemic medical conditions and medications:

Chronic systemic diseases may compromise wound healing and increase susceptibility to inflammation. In individuals with poorly controlled diabetes, altered collagen metabolism and elevated infection risk can delay gingival healing and reduce the predictability of restoration margins. Autoimmune conditions such as Sjögren's syndrome reduce salivary gland secretion, contributing to mucosal dryness and lowered tissue resilience [31]. Medications also play an important role. Agents such as anticonvulsants, immunosuppressants and calcium-channel blockers are known to induce gingival enlargement, making plaque control more difficult and potentially influencing the placement and contour of restoration margins [32]. Antiresorptive drugs, including bisphosphonates, have been associated with medication-related osteonecrosis of the jaw, which can severely impair soft tissue healing following dental procedures [33].

## Xerostomia and soft tissue integrity:

Adequate saliva is essential for lubrication, antimicrobial defense and buffering of acids. Xerostomia, whether related to systemic disease or medication use, can lead to fragile mucosa, burning sensations and increased susceptibility to infections. Importantly, xerostomia has been associated with a significantly higher rate of restoration failure and tooth loss, highlighting the importance of evaluating salivary flow before planning restorative treatment [34].

## Oral hygiene and smoking:

Plaque accumulation is a major contributor to gingival inflammation and soft tissue breakdown around restorations. Poor oral hygiene can result in bleeding, recession and attachment loss, compromising the esthetics and longevity of restorative work. Smoking further intensifies these effects by impairing vascular perfusion and immune function, leading to an increased risk of gingival recession and marginal tissue deterioration [35].

## Clinical implications:

These factors often interact. For example, a patient with diabetes who also smokes and is taking xerostomia-inducing medication may experience delayed healing, reduced tissue tolerance and difficulty maintaining oral hygiene. A comprehensive evaluation, including medical history, medication review and assessment of salivary function, enables clinicians to tailor preventive and restorative strategies, improving healing potential and overall treatment predictability [36].

## Conclusion:

Patient-specific medical and behavioural factors profoundly shape soft tissue outcomes in restorative dentistry. Clinical success in restorative dentistry hinges on several key procedures, with soft tissue management playing a pivotal role. Addressing these variables through risk assessment and tailored interventions enables more predictable, esthetic and durable restorative results.

## References

[1] Muscholl C (2022). J Adhes Dent..

[2] Gopalakrishnan D (2024). J Indian Soc Periodontol..

[3] Thoma DS (2022). Periodontol 2000..

[4] Alrmali A (2024). Clin Exp Dent Res..

[5] El-Maáaita AM (2024). J Adhes Dent..

[6] Mulla SA (2023). Cureus..

[7] Katariya C, Rajasekar A (2024). Cureus..

[8] Siegenthaler M (2022). J Clin Periodontol..

[9] Ferencz JL (1991). J Prosthet Dent..

[10] Dietschi D, Spreafico R (1998). Pract Periodontics Aesthet Dent..

[11] Panikkar P (2022). Int J Health Sci..

[12] Nugala B (2012). J Conserv Dent..

[13] John P (2015). J Interdiscip Dent..

[14] Zhang X (2020). Int J Oral Sci..

[15] Adnan S, Agwan MA (2018). Dental Update..

[16] Safari S (2016). J Dent Biomater..

[17] Donovan TE, Chee WW (2004). Dent Clin North Am..

[18] Kazakova R (2023). Healthcare (Basel)..

[19] Baba NZ (2014). Dent Clin North Am..

[20] Sampath P (2019). Indian J Dent Sci..

[21] Verma SK (2012). Natl J Maxillofac Surg..

[22] Babaji P (2014). J Pediatr Dent..

[23] Mehta S (2019). Contemp Clin Dent..

[24] Alqahtani SM (2022). World J Dent..

[25] Kavita K (2020). J Pharm Bio allied Sci..

[26] Hausdörfer T (2024). Clin Oral Investig..

[27] Al Baker AM (2015). J Contemp Dent Pract..

[28] Desclos-Theveniau M (2025). J Prosthet Dent..

[29] Alhamdan MM (2024). Cureus..

[30] Thomson WM (2015). Aust Dent J..

[31] Fox PC (2007). Acad Sci..

[32] Trackman PC, Kantarci A (2004). Crit Rev Oral Biol Med..

[33] Ruggiero SL (2014). J Oral Maxillofac Surg..

[34] Diab E (2021). Eur Arch Paediatr Dent..

[35] Bergström J (2004). Odontology..

[36] Kusumadewi S (2025). Intisari Sains Medis..

